# Running Economy After a Low‐ and High‐Intensity Training Session in Naturally Menstruating Endurance‐Trained Female Athletes: The FENDURA Project

**DOI:** 10.1111/sms.70050

**Published:** 2025-04-04

**Authors:** Heleen Docter, Madison Taylor, Anna Lena Müller, Jos J. de Koning, Øyvind B. Sandbakk, John O. Osborne, Dionne A. Noordhof

**Affiliations:** ^1^ Department of Human Movement Sciences Vrije Universiteit Amsterdam the Netherlands; ^2^ Centre for Elite Sports Research, Department of Neuromedicine and Movement Science Norwegian University of Science and Technology Trondheim Norway; ^3^ School of Sport Sciences UiT The Arctic University of Norway Tromsø Norway; ^4^ Sunshine Coast Hospital and Health Service Birtinya Australia; ^5^ School of Health University of the Sunshine Coast Sippy Downs Australia; ^6^ School of Exercise and Nutrition Sciences Queensland University of Technology Brisbane Australia

**Keywords:** durability, energy cost, menstrual cycle, oxygen consumption, resilience

## Abstract

The ability to maintain running economy is generally evaluated during a long continuous exercise bouts, and it is unclear whether the menstrual cycle phase acts as a confounder. The first aim of this study was to evaluate the ability to maintain running economy during typical 1‐h low‐ (LIT) and high‐intensity training (HIT) sessions in female athletes. The second aim was to investigate whether menstrual cycle phase affected the ability to maintain running economy. Naturally menstruating endurance‐trained females performed three LIT (*n* = 16) (45%–55% of the maximal velocity achieved during the maximal incremental test) and/or three HIT sessions (*n* = 17) (5 × 4 min at 80% of the maximal velocity achieved during the maximal incremental test) during three distinct menstrual cycle phases: early follicular, ovulatory, and mid luteal. Running economy was determined before and after each session. Running economy, expressed as energy cost (before: 1.34; after: 1.34 kcal/kg/km, *p* = 0.797) and oxygen cost (before: 272, after: 273 mL/kg/min, *p* = 0.348), was not significantly different before versus after the LIT session. Energy cost (before 1.33; after: 1.34 kcal/kg/km, *p* = 0.130) was not significantly different before versus after the HIT session, but oxygen cost (before: 269; after: 274 mL/kg/km, *p* < 0.003) was slightly higher after the session. Menstrual cycle phase did not confound the ability to maintain running economy. Running economy can be maintained during a typical 1‐h LIT session. The ability to maintain running economy during a typical HIT session depends on the expression used; energy cost was unaffected, while oxygen cost may be slightly increased after HIT sessions.

## 1 Introduction

Maximal oxygen uptake (V̇O_2max_), accumulated oxygen deficit, and gross efficiency/economy are considered important determinants of endurance performance [1]. However, these variables may change from their initial “unfatigued state” value during prolonged or fatiguing exercise. The ability to maintain these performance‐determining variables stable during prolonged or fatiguing exercise is commonly termed durability or resilience and is thought to be an important predictor of race performance [2].

Running economy has been shown to deteriorate during long efforts (e.g., ≥ 60 min) at a constant velocity above 60% of the maximal oxygen uptake (V̇O_2max_) [3, 4, 5, 6, 7, 8, 9]. The effect of short (< 60 min) constant velocity runs on running economy is contradictory. Constant velocity runs of 40 min at 80% V̇O_2max_ [6] did not alter economy, while a 5 km run at 80%–85% V̇O_2max_ and a trail run of 18.4 km with fluctuating intensity did impair running economy [7, 10, 11]. Therefore, exercise with a duration ≥ 60 min and an intensity > 60% V̇O_2max_ likely impairs running economy, although it is currently unclear whether running economy deteriorates during exercise of shorter duration and/or lower intensities.

Fatiguing exercise protocols used in durability studies often differ from typical training sessions performed by endurance athletes. Approximately 75%–80% of the total training duration of endurance athletes consists of low‐intensity training (LIT) and approximately 5%–10% consists of high‐intensity training (HIT) sessions [12, 13]. Typical LIT sessions are performed at intensities below the first lactate threshold while typical HIT sessions consist of high‐intensity intervals (above the second lactate threshold; onset of blood lactate accumulation) alternated with recovery periods [13]. Running economy changes following typical LIT and HIT sessions are, to the best of our knowledge, not yet examined.

Males and females may differ in their ability to maintain the performance‐determining variables throughout fatiguing exercise [8, 10, 14, 15]. Males and females differ, among others, in their hormonal environment. Naturally menstruating females have fluctuating concentrations of estrogen and progesterone across a 21–35‐day menstrual cycle. As estrogen and progesterone have been found to influence multiple physiological systems, the changing hormonal levels across the cycle have the potential to affect training responses [16]. Due to inconsistencies in the existing literature, it is unclear if and how menstrual cycle phase influences running economy in an unfatigued state [14, 15, 17, 18]. Lee et al. [19] reported compromised durability in the luteal phase compared to the follicular phase of the menstrual cycle. In their study, durability was evaluated as the stability in mean cycling power during repeated high‐intensity (6 × 1 min) efforts, before and after 45 min moderate‐ and 6 min high‐intensity cycling. Although running economy has been found to strongly correlate with performance [20], we are unaware of studies that evaluated the influence of menstrual cycle phase on the ability to maintain running economy, as a measure of durability, throughout a standardized LIT and HIT session.

Therefore, the first aim of this study was to evaluate the ability to maintain running economy during a typical LIT and HIT session in endurance‐trained females. The second aim was to investigate whether menstrual cycle phase affects the ability to maintain running economy. For this purpose, LIT and HIT sessions were performed in three hormonally distinct menstrual cycle phases: early follicular (EF), ovulatory (O), and mid luteal (ML) phase [21]. We hypothesized that running economy would remain stable during the LIT session but deteriorate during the HIT session. We expected the deterioration in running economy during the HIT session to be compromised in the ML phase, as performance has been shown to have declined in the ML phase during cycling [19].

## 2 Methodology

### 2.1 Study Design

Running economy was determined before and after typical LIT and HIT sessions, which were performed in three distinct menstrual cycle phases. Prior to the test period, participants were tracked for up to two menstrual cycles using calendar‐based counting and at‐home urinary ovulation kits (Clearblue Digital Ovulation kits, Swiss Precision Diagnostics GmbH, Geneva, Switzerland). During the early follicular phase (EF, days 1–4 from the onset of menses) of one lead‐in cycle, participants performed a running lactate profile and maximal incremental running test on a treadmill to determine their exercise capacity. During the subsequent two menstrual cycles, participants performed three supervised LIT or HIT training sessions during the EF phase, O phase (within 36 h after a positive urinary ovulation test) and ML phase (7–9 days after the day of a positive urinary ovulation test) [21]. Participants were randomly allocated to start with LIT or HIT testing and repeated the same session in the three consecutive phases of a single menstrual cycle. To increase recruitment of potential participants, testing was conducted at three testing locations across Norway (Tromsø, UiT The Arctic University of Norway; Trondheim, Norwegian University of Science and Technology; and Oslo, Norwegian School of Sport Science), with identical testing procedures.

The current study was part of the Female Endurance Athlete (FENDURA) project, as previously described [22]. Ethical approval for the study was waived by the Regional Committee for Medical and Health Research Ethics (REK, Project‐ID: 230505). The study was preapproved by the Norwegian Social Science Data Services (NSD, Project‐ID: 955558) and performed in accordance with the institutional ethical requirements and the Declaration of Helsinki. All participants received written and oral information prior to enrolling in the study and provided written informed consent to participate.

### 2.2 Participants

Participants were recruited through local sporting clubs, personal connections, and social media. The prescreening criteria were as follows: (1) females, aged 17–40 years old, (2) reported having a regular menstrual cycle length of 21–35 days over the preceding 3 months, (3) systematically trained in an endurance sport at least three times per week and/or 5 h per week, (4) did not use hormonal contraceptives for at least 3 months prior to the onset of the study, (5) reported no clinically defined menstrual disorder (i.e., polycystic ovarian syndrome), (6) were not pregnant or trying to get pregnant, and (7) had no injuries or illness preventing the participant from training regularly.

**FIGURE 1:**
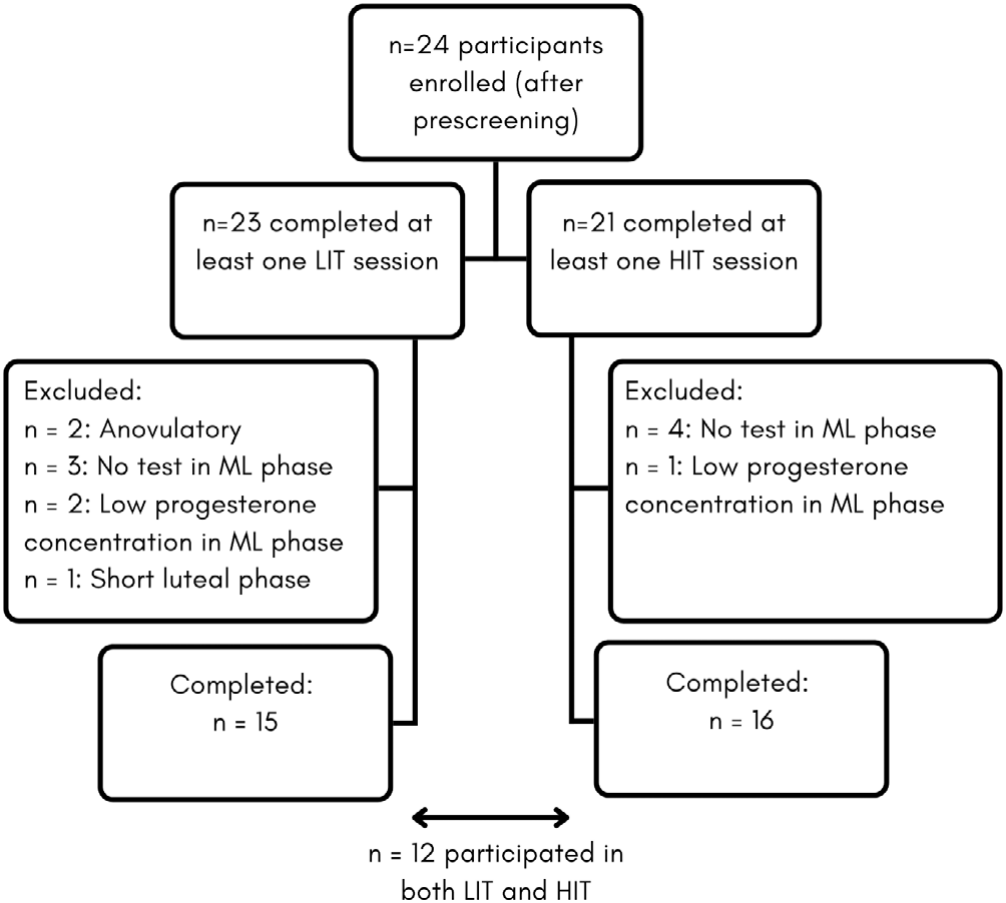
Participant inclusion for the low‐ and high‐intensity training session. HIT = high‐intensity training, LIT = low‐intensity training, and ML = mid luteal.

After prescreening, 24 eligible participants were enrolled in the study. Menstrual cycle phases were verified using the 3‐step method [23], including calendar‐based counting, urinary ovulation testing, and serum hormone analysis of estrogen and progesterone, as previously described by Taylor et al. [18]. Data were retrospectively excluded from the analysis if the test cycle was anovulatory, progesterone concentration was < 16 nmol/L in the ML phase [21, 24] or the luteal phase was shorter than 10 days [18, 21] (Figure 1). If participants missed a test in the ML phase, data of that menstrual cycle were excluded as screening for luteal phase deficiencies could not be performed.

Participants were instructed to record their nutritional intake during the 24 h before the lead‐in test using an online diary and to replicate this diet before each subsequent test session. To minimize the influence of menstrual cycle phase on glycogen storage [25], participants were provided with individualized dietary recommendations to encourage a carbohydrate intake of at least 8 g per kg body mass before their testing sessions [26]. Participants were asked to refrain from intensive exercise in the last 24 h and caffeine intake in the 8 h prior to each test. Throughout the study period, participants were asked to maintain their weekly exercise routine. Participants logged all exercise activities in the online platform “Olympiatoppens Treningsdagbok” the Norwegian Top Sports Center (Olympiatoppen) training diary, or BESTR training diary (BESTR). Average hours per week and sessions per week were calculated retrospectively over a period of 28 days before the supervised exercise session in the ML phase. Mobility or stretching exercises were not included.

#### 2.2.1 Protocol

##### 2.2.1.1 Lead‐In Period

In the EF phase of the lead‐in period, a lactate profile test and a maximal incremental exercise test were performed running on a treadmill (Woodway PPS Med 55, Waukesha, Wisconsin, USA) with an incline of 5% to establish V̇O_2max_ and the maximal velocity achieved during the maximal incremental test (*v*_max_). Participants warmed up for 5 min at an intensity of approximately 50% of their self‐reported maximal heart rate. The lactate profile test started at a velocity of 7–10 km/h, dependent on the expected fitness level of the participant. Every 5 min, velocity was increased by 1 km/h until a blood lactate concentration > 4 mmol/L or a RPE of 17 was reached. After 10 min of rest, the participants started the maximal incremental test at a velocity of two steps lower than the last step of the lactate threshold test, followed by an increase in velocity of 1 km/h per minute until exhaustion. Gas exchange variables were monitored using the Jaeger Vyntus CPX (Vyaire medical GMBH, Höchberg, Germany) and a two‐way breathing valve (2730 series, Hans Rudolph Inc., Kansas City, MO, USA). The Vyntus CPX was used in mixing chamber mode with a sampling period of 5 s, and data were recorded using SentrySuite software (Vyaire medical GMBH, Höchberg, Germany).

##### 2.2.1.2 Test Day Protocol

**FIGURE 2:**
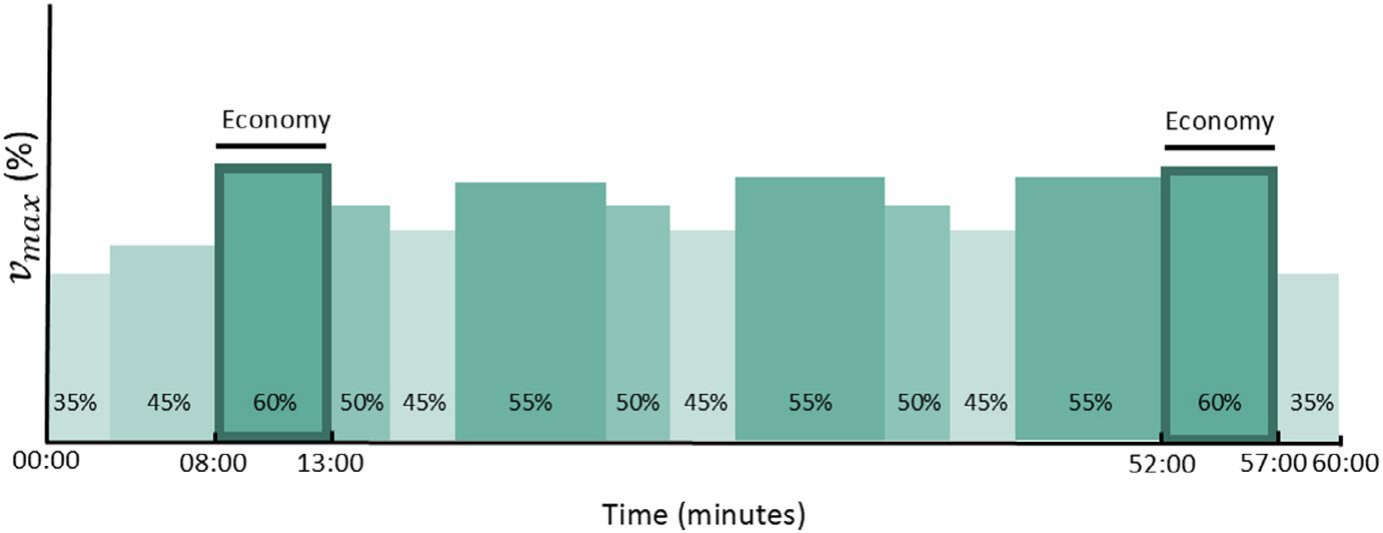
Visual representation of the low‐intensity training session. Running economy was determined during the highlighted blocks at 60% *v*_max_. *v*_max_ = maximal velocity achieved during the maximal incremental test.

**FIGURE 3:**
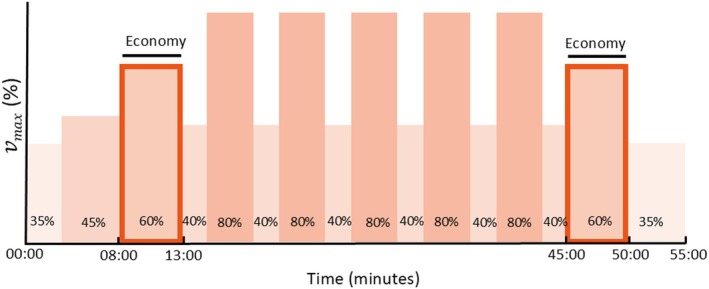
Visual representation of the high‐intensity training session. Running economy was determined during the highlighted blocks at 60% *v*_max_. *v*_max_ = maximal velocity achieved during the maximal incremental test.

Upon arrival at the laboratory, a venous blood sample was collected in a fasted state (between 6:00 a.m. and 10:00 a.m.) to verify menstrual cycle phases [18]. Subsequently, the participant received a standardized low‐fiber breakfast consisting of 2 g carbohydrate/kg body mass (i.e., banana, bread, jam, and chocolate spread). After a 90‐min rest period, participants performed a standardized warm‐up on the treadmill (3‐min at 35% *v*_max_, 5‐min at 45% *v*_max_), followed by 5 min of running at an intensity of 60% *v*_max_ to determine running economy before the session. After the economy block, participants started the main set of the LIT or HIT session. The main set of the LIT session consisted of three blocks of 3 min at 50% *v*_max_, 3 min at 45% *v*_max_, and 7 min at 55% *v*_max_ (Figure 2). The main set of the HIT session consisted of 5 × 4 min at 80% *v*_max_, with 2 min of recovery at 40% *v*_max_ (Figure 3). If 80% *v*_max_ could not be completed during the first interval of the first test, exercise intensity was scaled down 5% for consecutive intervals, and the same procedure was repeated during consecutive test sessions. Immediately after the main set of the LIT and HIT session, participants repeated the 60% *v*_max_ block to determine running economy after the session. The warm‐up, economy blocks, and main set of the LIT and HIT session resulted in a session duration of 60 and 55 min, respectively. Treadmill incline was 5% during all training sessions. Gas exchange variables were monitored during the 60% *v*_max_ blocks to determine running economy before and after the LIT and HIT session. Session RPE was rated on a Borg scale (6–20) [27] after completion of the session. The average velocity of the 60% *v*_max_ blocks was 8.5 ± 0.8 km/h.

### 2.3 Data Analysis

Speed at onset of blood lactate accumulation was defined as the speed when lactate concentration reached 4 mmol/L, determined by interpolation. Data from the maximal incremental test was used to determine V̇O_2max_, defined as the highest oxygen consumption (V̇O_2_) over a 30‐s moving average. *v*_max_ was defined as the highest velocity achieved during the maximal incremental test (last completed step). In case of an incomplete step, the average velocity was calculated based on the percentage of time completed.

Respiratory data of the LIT and HIT sessions were used to determine the average V̇O_2_ and RER from minute 2:50 till 4:50 of the 5‐min submaximal exercise blocks at 60% *v*_max_ before and after the LIT and HIT sessions. Running economy was expressed as energy cost (EC) at a given velocity (EC in kcal/kg/km) and oxygen cost at a given velocity (OC in mL/kg/km) [28]. EC was calculated via the metabolic rate, by multiplying V̇O_2_ (L/s) by the oxygen equivalent [29]:

$$\text{metabolic rate}=\dot{V}O_{2}∙\frac{\left(4940∙\mathrm{RER}+16040\right)}{\text{body mass}}$$

Metabolic rate (W/kg) was divided by running speed (in m/s) and subsequently converted from Watts to kilocalories (1 kcal = 4184 J) [30] to get a value for EC. Body mass was determined immediately before each training session. OC was calculated based on V̇O_2_ (mL/min), body mass (kg), and running speed [17]. To ensure steady state, EC and OC values were accepted if the average RER did not exceed 1.00 and the difference in mean V̇O_2_ over the first and last 30 s of the analyzed time frames was ≤ 10%. The average %V̇O_2max_ during the last 3 min of the 5‐min block at 55% *v*_max_ (LIT) and 4‐min blocks at 80% *v*_max_ (HIT) was calculated to have an indication of the physiological strain of the training session. Calculations were made using Matlab (MathWorks Inc., Natick, USA).

### 2.4 Statistics

Statistical analyses were performed using R in the RStudio environment [31]. The ability to maintain running economy (EC and OC) during a LIT or HIT session was modeled with mixed linear regressions (package “lme4,” version 1.1.33). The timing of the economy determination was added as a fixed effect (two levels: before, after). A random intercept for each participant was added to correct for repeated measures. Similar models were used to investigate the ability to maintain V̇O_2_ and RER throughout a LIT or HIT session (fixed effect with two levels: before, after; random intercept for participant). A separate model was used to check whether menstrual cycle phase acted as a confounder in the ability to maintain EC. Menstrual cycle phase (three levels: EF, O, and ML) was added as a covariate in the LIT and HIT model and was recognized as a confounder when the regression coefficient of the fixed effect changed by more than 10% [32]. Only EC was used in this model as it is a measure of the energy cost (not oxygen cost) that is often used as a unit for running economy. Although mixed models are robust to violations of model assumptions [33], all models were checked for homoscedasticity and normality of the residuals, and no potential problems were identified. Marginal means, confidence intervals, and Cohen's D were derived for each model using the “emmeans” package (version 1.8.5) [34]. Conditional and marginal R^2^ were calculated using the “performance” package (version 0.12.0) [35]. The level of significance was set to *p* = 0.05. Figures were generated using “ggplot2” (version 3.4.2) [36].

## 3 Results

Fifteen participants were included in the LIT analysis (mean ± standard deviation: maximal oxygen consumption [V̇O_2max_] = 52.7 ± 10.6 mL/kg/min, speed at onset of blood lactate accumulation = 10.3 ± 1.3 km/h, height 168 ± 5 cm, and body mass 63 ± 6 kg) and 16 participants were included in the HIT analysis (V̇O_2max_ = 53.1 ± 10.4 mL/min, speed at onset of blood lactate accumulatio*n* = 10.4 ± 1.4 km/h, height 168 ± 5 cm, and body mass 62 ± 7 kg). Thirteen participants participated in both the LIT and the HIT sessions. The 55% *v*_max_ blocks during the LIT session corresponded to approximately 66% ± 5% of the participants' V̇O_2max_. The 80% *v*_max_ during the LIT session corresponded to approximately 91% ± 5% of the participants' V̇O_2max_. Participants self‐reported a total exercise duration of 7 ± 3 h per week and frequency of 7 ± 3 sessions per week throughout the measurement period. Participants were classified as Tier 2 (*n* = 15), Tier 3 (*n* = 3), and Tier 4 (*n* = 1) [37].

**FIGURE 4:**
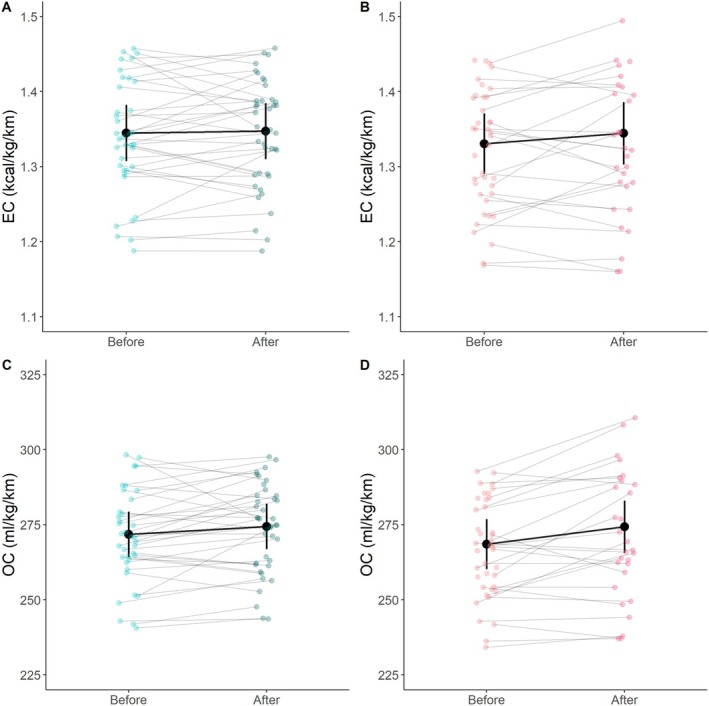
Mean values and confidence intervals of the energy cost per km (A and B) and oxygen cost (D and E), before and after the low‐ (left) and high‐ (right)intensity training session. Each test of each participant is represented by a small dot and line. *Represents a significant difference before versus after. EC = energy cost, OC = oxygen cost.

Running economy, expressed as EC and OC, was not significantly different before versus after the LIT session (*p =* 0.138 and *p* = 0.766, respectively, Table 1, Figure 4A,C). V̇O_2_ was also not significantly different before versus after the LIT session (*p =* 0.100, Table 1), while RER was significantly lower after the LIT session (*p <* 0.001, Table 1). Median session RPE for the LIT session was 11 (range: [7–14]).

**TABLE 1:** Energy cost (EC), oxygen cost (OC), oxygen consumption (V̇O_2_), and respiratory exchange ratio (RER) before and after low‐ and high‐intensity training sessions by female endurance athletes.

	Low‐intensity training								High‐intensity training							
	Before	After	n	*χ* ^2^	*p*	*R* ^2^ conditional	*R* ^2^ marginal	Cohen's d	Before	After	n	*χ* ^2^	*p*	*R* ^2^ conditional	*R* ^2^ marginal	Cohen's d
EC (kcal/kg/km)	1.34 [1.31, 1.38]	1.35 [1.31, 1.38]	15	0.092	0.762	0.77 [0.77, 0.87]	0.00 [0.00, 0.02]	−0.01	1.33 [1.29, 1.37]	1.34 [1.30, 1.39]	16	2.29	0.130	0.81 [0.79, 0.91]	0.01 [0.00, 0.03]	−0.39
OC (mL/kg/km)	272 [264, 279]	274 [267, 282]	15	2.316	0.128	0.74 [0.71, 0.86]	0.01 [0.00, 0.04]	−0.03	269 [260, 277]	274 [266, 283]*	16	8.86	0.003	0.81 [0.82, 0.91]	0.03 [0.00, 0.09]	−0.78
V̇O_2_ (mL/kg/min)	37.6[36.3, 38.9]	38.0[36.7, 39.3]	15	2.86	0.091	0.82 [0.79, 0.90]	0.01 [0.00, 0.03]	−0.38	2383 [2237, 2530]	38.1 [36.5, 39.7]*	16	15.6	< 0.001	0.90 [0.89, 0.95]	0.02 [0.01, 0.05]	−0.86
RER	0.91 [0.89, 0.94]	0.88 [0.85, 0.91]*	15	69.8	< 0.001	0.87 [0.86, 0.92]	0.11 [0.07, 0.18]	1.84	0.92 [0.89, 0.94]	0.86 [0.83, 0.88]*	16	265	< 0.001	0.90 [0.89, 0.95]	0.30 [0.22, 0.36]	3.44

*Note:* Data presented as estimated marginal means (95% confidence interval).

Abbreviations: EC = energy cost, *n* = number of participants with data, OC = oxygen cost, RER = Respiratory Exchange Ratio, V̇O_2_ = oxygen consumption.

* Represents a significant pre–post difference (*p <* 0.05).

Running economy, expressed as EC, was not significantly different before versus after the HIT session (*p =* 0.130, Table 1, Figure 4B). However, running economy, expressed as OC, was 1.8% higher (*p =* 0.003, Table 1, Figure 4F) after completion of the HIT session compared to before. V̇O_2_ was 2.4% higher (*p <* 0.001, Table 1), while RER was 6.5% lower after the HIT session compared to before (*p <* 0.001, Table 1). Median session RPE for the HIT session was 16 (range: [14–19]).

**FIGURE 5:**
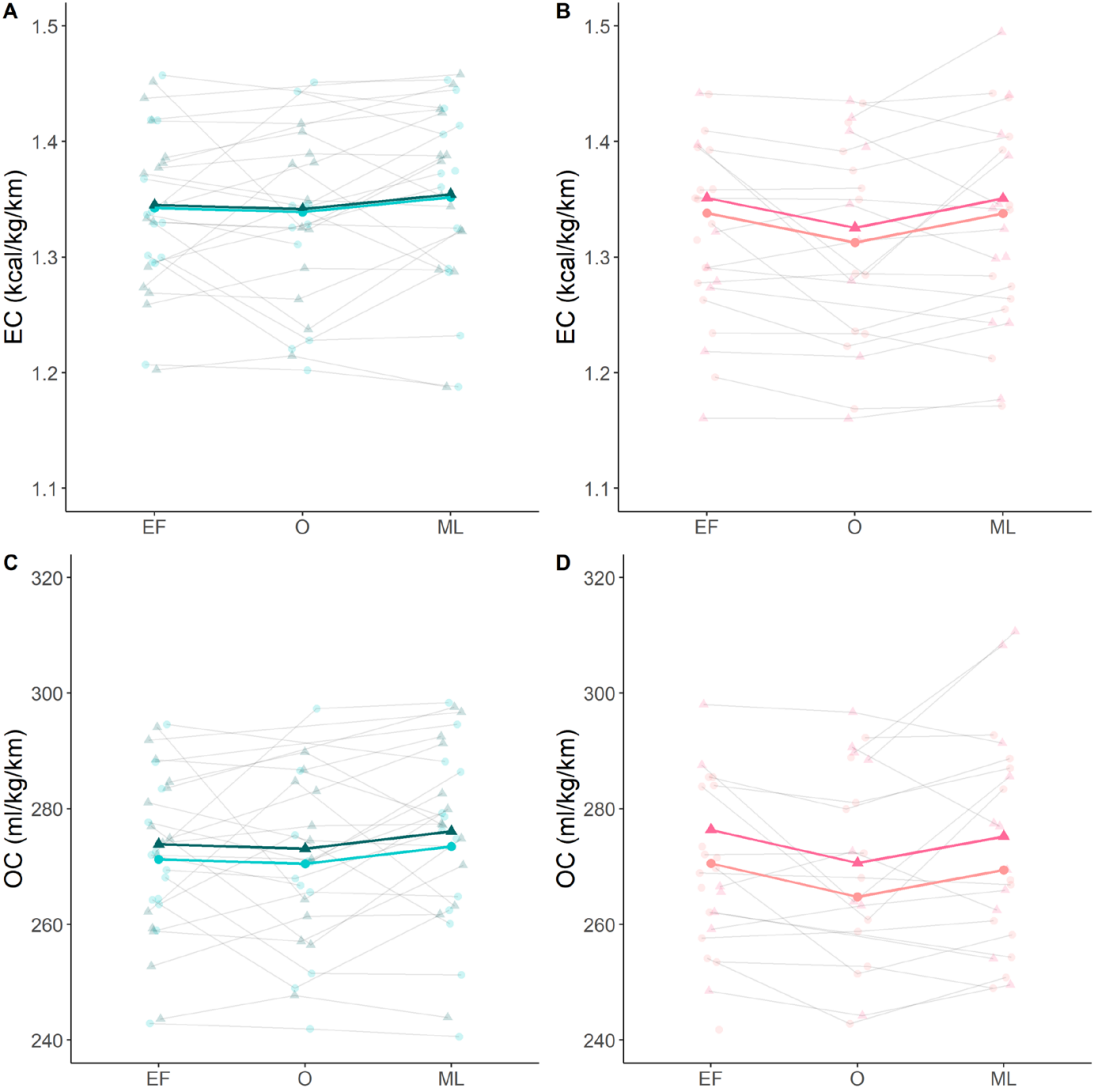
Mean values of the energy cost per km (A and B) and oxygen cost (D and E), before (dots) and after (triangles) the low‐ (left) and high‐ (right)intensity training session. Each test of each participant is represented by a small dot and line. *Represents a significant difference before versus after. EC = energy cost, EF = early follicular, ML = mid luteal, O = ovulatory, OC = oxygen cost.

Menstrual cycle phase was not a confounding factor for the ability to maintain EC during a LIT or HIT session, as the regression coefficients changed < 10% (Figure 5).

## 4 Discussion

The current study evaluated running economy before and after a typical LIT and HIT session in naturally menstruating endurance‐trained females. Furthermore, we investigated the potential confounding effect of menstrual cycle phase on the ability to maintain running economy throughout the standardized training sessions. The main findings were that (1) running economy was unaffected by the LIT and HIT sessions when economy was expressed as EC, while economy deteriorated during the HIT session when it was expressed as OC, and (2) menstrual cycle phase did not act as a confounder on the ability to maintain economy.

### 4.1 Low‐ and High‐Intensity Training Session

Running economy, expressed as EC and OC, was not significantly different before versus after the LIT session, which is in line with our hypothesis. A 60‐min run at 45%–55% *v*_max_ did not seem to exert enough physiological strain on our participants to deteriorate running economy.^38^ In contrast to our hypothesis, running economy, expressed as EC, was not significantly different before versus after the HIT session. OC and V̇O_2_ were, respectively, 1.8% and 2.4% higher after the HIT session. The practical relevance of a change in OC and V̇O_2_ depends, among others, on the within‐subject variation. Previous research has reported a small worthwhile change (0.2* between‐subject standard deviation) of 2.2%–2.6% and a coefficient of variation of 2.5%–2.9% (within‐subject) for V̇O_2_ in highly trained distance runners during running at 14–18 km/h [38, 39]. Although the within‐participant coefficient of variation was obtained on different testing days, the reference values could help to put the changes in V̇O_2_ into perspective. An increase in V̇O_2_ of 2.4% as found during the HIT session falls just below the range reported for within‐subject variation. Therefore, the practical relevance for runners is questionable.

Interestingly, the method by which running economy is measured or expressed seems to influence the outcome. Several studies reported a deteriorated running economy (in mL/kg/m or mL/kg/min) after 40–60‐min constant velocity runs at 60%–80% *v*_max_ [3, 4, 5, 6, 8, 9, 28], or after simulated races [7, 10, 11, 40], but only two of these studies expressed economy as EC [28, 40]. OC (mL/kg/km) differs conceptually from EC (in kcal/kg/km), as it represents the oxygen demand instead of the energy demand [28]. EC involves V̇O_2_ and RER in its calculations [29], and arguably provides a more precise representation of the physiological demand [28]. Zanini et al. [28] found a significant deterioration of EC and OC after a 90‐min run at approximately 80% V̇O_2max_, but the deterioration was smallest when running economy was expressed as EC. Besson et al. [40] reported a deterioration in running economy (J/kg/km) after ultra trail running events of various lengths, for example, 40 km, 56 km, 101 km, 145 km, and 171 km. Both Zanini et al. [28] and Besson et al. [40] used longer running durations and/or higher exercise intensities than the current study. Therefore, EC may deteriorate if running distance or duration were to increase but remained unaffected during the 60‐min LIT and HIT sessions.

The current study differs from existing literature with respect to the low intensity of the LIT session and intermittent nature of the HIT session. Previous research on low‐intensity efforts has involved longer durations (40 or 56 km) [40] or higher intensity runs (≥ 60% V̇O_2max_) [3, 4, 5, 6, 7, 8, 9, 10, 11, 28] than the LIT session of the present study. Our study suggests that a duration of 60 min is insufficient to impair running economy during low‐intensity runs. Only two other studies have evaluated economy (mL/kg/km) during high intensity efforts, both after an 18.4‐km trail run with a duration of ~110 min across varying terrain and found a deterioration in running economy [10, 11]. The physiological demands imposed by the varying terrain differ from the typical HIT session used in this study. The HIT intervals are intended to be performed at an intensity above the second lactate threshold (i.e., onset of blood lactate accumulation) to induce an inability to achieve intramuscular metabolic homeostasis and hypothetically lead to a deterioration in economy (or lower efficiency) [41, 42]. The 2‐min rest period may have restored homeostasis and thereby preserved running economy. Literature suggests that 2 min of rest provides an additional performance benefit compared to 1 min of rest during 5 × 4 min running intervals, while a 4‐min rest period does not provide additional performance benefits when evaluated as mean running speed [43]. Further research should potentially investigate if certain interval‐rest ratios can be used to preserve homeostasis.

Additionally, the current body of literature involves predominantly male participants, while our study only included female participants. There is some evidence that running economy is more prone to deteriorate after fatiguing exercise in male participants compared to female participants [9]. Less homeostatic disturbances [44] and higher rates of recovery are found in females compared to males [45]. Inclusion of a male participant group would enable comparison of male and female responses and evaluate whether the ability to maintain running economy during typical LIT and HIT training sessions is a female‐specific finding.

Some participants may be more vulnerable to changes in running economy than others. Interindividual variability in durability of 1%–32% has been reported in cycling, when durability was evaluated as critical power [2]. Additionally, the method used to prescribe exercise intensity (as % *v*_max_) may have introduced additional variation in physiological responses between participants. The speed of the interval sessions was prescribed as 80% of the participants' *v*_max_, which fell below the lactate threshold (lactate > 4 mmol) for four out of 17 participants. The variety in exercise intensity relative to the lactate threshold may have introduced variation in the fatiguing effect and thereby limited the deterioration in economy at a group level. Visual inspection revealed that economy did not consistently drop in the participants that reached the desired exercise intensity. Therefore, exercise intensity cannot fully explain why we did not find a change in running economy expressed as EC.

### 4.2 Menstrual Cycle Phase Not a Confounder

The ability to maintain running economy after exercise was not affected by menstrual cycle phase. We hypothesized that the ability to maintain running economy would be compromised in the ML phase, based on previous research reporting a larger drop in cycling power output during repeated sprints in the luteal compared to the follicular phase [19]. We evaluated running economy instead of cycling power output, which complicates direct comparisons between the studies. However, we are unaware of other studies that evaluated the influence of menstrual cycle phase on running economy as a measure of durability. Previous studies have investigated the effect of menstrual cycle phase on running economy in a fresh state, but findings are inconclusive. Economy has been shown to be lower in the follicular phase [17], lower in the mid luteal phase [14, 15] or unaffected by menstrual cycle phase [18]. These conflicting findings may be explained by small sample sizes, high individual variation reported in menstrual cycle‐related studies [18], differences in menstrual cycle phase definitions, and inadequate menstrual cycle phase verification [15, 17].

It is important to note that we evaluated the confounding effect of menstrual cycle phase using three distinct menstrual cycle phases. The EF, O, and ML phases were determined and verified using the 3‐step‐method, consisting of calendar‐based counting, urinary ovulation testing, and retrospective blood hormonal analysis [23]. It is possible that the selected menstrual cycle phases are not consistent with the time frames during which a meaningful change in economy is present. Moreover, further research is needed to evaluate whether the durability of running economy is also unaffected during longer LIT sessions. Based on our current findings and the existing evidence, it seems that the effect of menstrual cycle phase on durability, assessed using running economy, is likely minor.

### 4.3 Methodological Considerations

A potential limitation of the present study is that the LIT and HIT sessions were not designed to make direct comparisons (i.e., matched for total work performed or other variables). As a result, the effect of time (before vs. after) on LIT and HIT sessions was tested using separate regression models, and the outcomes were interpreted independently. It would be interesting to investigate whether the ability to maintain running economy is solely determined by the amount of work performed or also affected by the intensity and/or duration of the exercise bout. Knowledge about the interplay between intensity, duration, and the amount of work performed is important to understand if/how running economy is stressed during a normal LIT, moderate intensity training, and HIT session, to develop better strategies for training durability.

Additionally, the LIT session may have been too short in duration to provoke changes in running economy, due to a relatively low fatigue induced. Durations of 30–105 min (excluding warm‐up and cooling down) have been reported for running LIT sessions performed by Norwegian long‐distance runners [13]. Although a duration of 60 min can be one of the typical LIT running sessions of endurance athletes [13], it would be interesting to investigate LIT sessions of longer durations.

### 4.4 Perspective

Our findings suggest that the typical 60‐min HIT and LIT sessions utilized in this study do not result in an observable decline in running economy, expressed as EC, at a group level. However, a deterioration in OC was reported after the HIT session. This is particularly relevant for female athletes and their coaches who may be utilizing similar training sessions with the aim of improving durability. As variability in the ability to maintain running economy has been found within a relatively homogeneous participant group, care should be taken when applying our findings on an individual level. Moreover, menstrual cycle phase may be one of the numerous factors that influence physiological demands during exercise on an individual level (e.g., sleep, nutrition, previous training load, mood, and stress levels), but it does not seem to have a dominant role on a group level. From a research perspective, the findings of this study argue for a more general inclusion of females in research. Menstrual cycle phase does not confound the ability to maintain running economy during 60‐min LIT and HIT sessions, which means that there is no evidence to exclude females a priori from further research within this area.

## 5 Conclusions

Running economy was evaluated before and after a typical LIT and HIT training session with a duration of approximately 60 min. No changes in OC (in mL/kg/km) or EC (kcal/kg/km) have been observed after the LIT session. Running economy expressed as OC was slightly higher after the HIT session, but these differences were absent when running economy was expressed as EC. Menstrual cycle phase did not have a confounding effect on the ability to maintain running economy during a LIT and HIT session.

## Ethics Statement

Ethical approval for the study was waived by the Regional Committee for Medical and Health Research Ethics (REK, Project‐ID: 230505). The study was preapproved by the Norwegian Social Science Data Services (NSD, Project‐ID: 955558) and performed in accordance with the institutional ethical requirements and the Declaration of Helsinki. All participants received written and oral information prior to enrolling in the study and provided written informed consent to participate.

## Conflicts of Interest

The authors have no conflicts of interest to declare. All coauthors have seen and agree with the contents of the manuscript and there are no financial interests to report. We certify that the submission is original work and is not currently under review for publication at another publisher.

## Data Availability

The data that support the findings of this study are available from the corresponding author upon reasonable request.
